# Controllable synthesis porous Ag_2_CO_3_ nanorods for efficient photocatalysis

**DOI:** 10.1186/s11671-015-0892-5

**Published:** 2015-04-21

**Authors:** Shenghui Guo, Jianxing Bao, Tu Hu, Libo Zhang, Li Yang, Jinhui Peng, Caiyi Jiang

**Affiliations:** State Key Laboratory of Complex Nonferrous Metal Resources Clean Utilization, Kunming University of Science and Technology, Kunming, 650093 China; National Local Joint Laboratory of Engineering Application of Microwave Energy and Equipment Technology, Kunming, 650093 China; Key Laboratory of Unconventional Metallurgy, Ministry of Education, Kunming, 650093 China; Faculty of Metallurgical and Energy Engineering, Kunming University of Science and Technology, No. 253 Xuefu Rd., Kunming, 650093 China

**Keywords:** Crystal growth, Porous nanorods, Silver carbonate, Photocatalysis

## Abstract

The novel porous Ag_2_CO_3_ nanorods were facilely synthesized via a one-pot aqueous solution reaction at room temperature. The crystalline phase and size distribution of the nanorods were determined by X-ray diffraction (XRD) and scanning electron microscopy (SEM), respectively. In addition, the porous feature of nanorods was confirmed by transmission electron microscopy (TEM) and nitrogen adsorption-desorption. The morphology and size of the Ag_2_CO_3_ crystal can be regulated via the choice of dispersing agents and adding approaches of reactants. Photocatalytic results show that the porous Ag_2_CO_3_ nanorods exhibit excellent photodegradation of rhodamine B (RhB) under visible-light irradiation, particularly the photoactivity performance and stability can be further improved in the presence of sodium bicarbonate (NaHCO_3_). It is indicated that NaHCO_3_ can prevent effectively the photocorrosion and promote the probability of electron-hole separation.

## Background

Semiconductor photocatalysts have attracted intense attention expecting to apply in the fields of pollution removal and fuel production by utilizing abundant sunlight [1-3]. Over the past years, TiO_2_ as the most universal used photocatalyst has been widely studied owing to its high photocatalytic activity, stability, nontoxicity, and low cost [4-7]. However, TiO_2_ is a wide bandgap (approximately 3.2 eV) semiconductor and difficult to be activated in visible-light region, only can be utilized under UV light which is a small fraction (about 4%) of the entire solar spectrum. In addition, TiO_2_ quantum yield of photoactivated processes is frequently lower due to its high recombination of photogenerated electron-hole pairs. Such clear drawback is the main motivation for searching a new, active under visible-light-driven and more efficient photocatalysts [8-10].

Recently, it has been found that silver-containing complex oxide semiconductors show great promise for improving photocatalytic performance owning to their tops of valence band can form a new higher-energy valence band consisting of the hybrid orbital of Ag 4d and O 2p, which make the bandgap narrower [11,12]. As a result, a series of visible-light-responsive novel silver-containing complex oxide semiconductor photocatalysts have been developed, such as AgNbO_3_ [13,14], AgSbO_3_ [12,15], Ag_2_CrO_4_ [16,17], Ag_2_SO_3_ [18], Ag_3_AsO_4_ [19], AgMO_2_ (M = Al,Ga,In) [20], and AgIO_4_ [21], and their active visible-light-driven photocatalysts for the degradation of organic pollutants have also been explored. More recently, it was reported that Ag_2_CO_3_ showed high-efficiency visible-light activity and exhibited universal degradation ability for typically several organic dyes [22-24]. However, the photocorrosion behavior of Ag_2_CO_3_ exposed to the light irradiation cannot be ignored. Hence, the addition of light stabilizer in reaction solution is critical to Ag_2_CO_3_ photocatalyst for its practical application. Dai et al. [23] prepared Ag_2_CO_3_ short rods by a simple precipitation reaction, and it showed that high visible-light photocatalytic activity for the photodegradation of rhodamine B (RhB). The authors stated that the silver nitrate (AgNO_3_) is beneficial to the stability during the photocatalytic degradation reaction process because it can act as an electron acceptor to avoid photocorrosion of Ag_2_CO_3_ photocatalyst. Moreover, it has been reported that the photocatalytic efficiency can be further improved by rational design to achieve porous structures, in that, the porous structures avail the adsorption of reactant molecules and provide multiple accessible passages which reduce the reactant diffusion distance due to their large specific surface area (SSA). Significantly, porous structure can produce more isolated and separated active sites after photoirradiation and provide special channels for charge transport, which results in high efficiency of charge separation and transport in under photoirradiation [25-27].

As far as we know, the synthesis of porous silver-containing complex oxide photocatalysts by one-pot aqueous solution reaction at room temperature has rarely reported. Herein, in the present work, we prepared a novel porous Ag_2_CO_3_ nanorod photocatalyst by one-pot aqueous solution reaction using PVP-K90 dispersing agent at room temperature. The as-prepared samples showed efficient photocatalytic activity for the degradation RhB aqueous solution by utilizing sodium bicarbonate (NaHCO_3_) as a light stabilizer under visible-light irradiations. Furthermore, the growth behavior of Ag_2_CO_3_ and photocatalysis enhanced mechanism of NaHCO_3_ were also discussed.

## Methods

### Materials

All the chemicals were analytic grade purity and were used without further purification. AgNO_3_, NaHCO_3_, polyvinylpyrrolidone (PVP-K30, PVP-K90) and RhB were purchased from Shanghai Chemical Regent Factory of China (Shanghai, China).

### Synthesis of porous Ag_2_CO_3_ nanorods

The porous Ag_2_CO_3_ nanorods were synthesized by a typically simple aqueous solution reaction at room temperature. In a typical synthesis, AgNO_3_ (0.025 M) and PVP-K90 (0.45 M) were dissolved in 40 mL deionized water to form a clear solution by magnetic stirring, then, 40 mL aqueous solution of NaHCO_3_ (0.05 M) was dropwise added to the obtained solution. The reaction was carried out at room temperature for 2 h under magnetic stirring, and the precipitate was collected by centrifugation, washed three times with deionized water and absolute ethyl alcohol, and dried at 50°C for 12 h. Furthermore, the synthesis of Ag_2_CO_3_ thin nanorods was similar to the above description except that PVP-K90 was replaced by PVP-K30. The cube-like Ag_2_CO_3_ was achieved by one-time injection of the NaHCO_3_ solution using PVP-K90 as the dispersing agent. N-doped TiO_2_, which is good photocatalytic activity under visible-light irradiation, was obtained as a reference to compare with our sample according to the reported literature [28].

### Characterization

Scanning electron microscopy (SEM) images were taken using a field-emission scanning electron microscope (JSM-6701 F, JEOL Ltd., Akishima-shi, Japan) and equipped with an energy-dispersive (ED) detector with this field-emission scanning electron microscope (FE-SEM) operated at 15 kV. Energy-dispersive X-Ray (EDX) analysis was also performed on the JSM-6701 F instrument during SEM. Transmission electron microscopy (TEM) images were obtained on a JEM-2100 electron microscope (JEOL Ltd., Akishima-shi, Japan) at an accelerating voltage of 200 kV. X-ray diffraction (XRD) data for the finely ground samples were collected at 298 K using a Bruker D8 X-ray diffractometer (Bruker AXS, Inc., Madison, WI, USA) with Cu-Kα radiation source (*λ* = 1.5406 Å). It was operated at 40 kV in the 2*θ* range of 10° to 80° in the continuous scan mode with the step size of 0.01°. The changes in the oxidation state of Ag were recorded though an AXIS-ULTRA DLD-600 W photoelectron spectrometer (Shimadzu Corporation, Kyoto, Japan) (XPS) with Al K1 radiation. Nitrogen adsorption-desorption isotherms were collected on an Autosorb-iQ sorption analyzer (Quantachrome Instruments, Boynton Beach, FL, USA) and analyzed followed by the Brunauer-Emmett-Teller (BET) equation. The pore size distribution plots were obtained by using the Barret-Joyner-Halenda (BJH) model.

### Photocatalytic performance measurements

The photocatalytic performance of the as-prepared samples was evaluated by measuring the degradation of RhB. In all catalytic activity of experiments, the samples (0.05 g) were put into a solution of RhB dyes (50 mL, 10 mg/L), which was then irradiated with a 300-W Xe arc lamp to provide visible light with *λ* ≥ 420 nm by an ultraviolet cutoff filter. Before the suspensions were irradiated, they were magnetically stirred for 30 min in the dark to complete the adsorption-desorption equilibrium between dyes and photocatalysts. The degradation of RhB was monitored by UV-vis spectrophotometer (UV-2550, Shimadzu Corporation, Kyoto, Japan) every 5 min. Before the spectroscopy measurement, these photocatalysts were removed from the photocatalytic reaction systems by centrifugation. The relative concentrations (*C*/*C*_0_) of the RhB solutions were determined by the absorbance (*A*/*A*_0_) at 553 nm.

## Results and discussion

**Figure 1:**
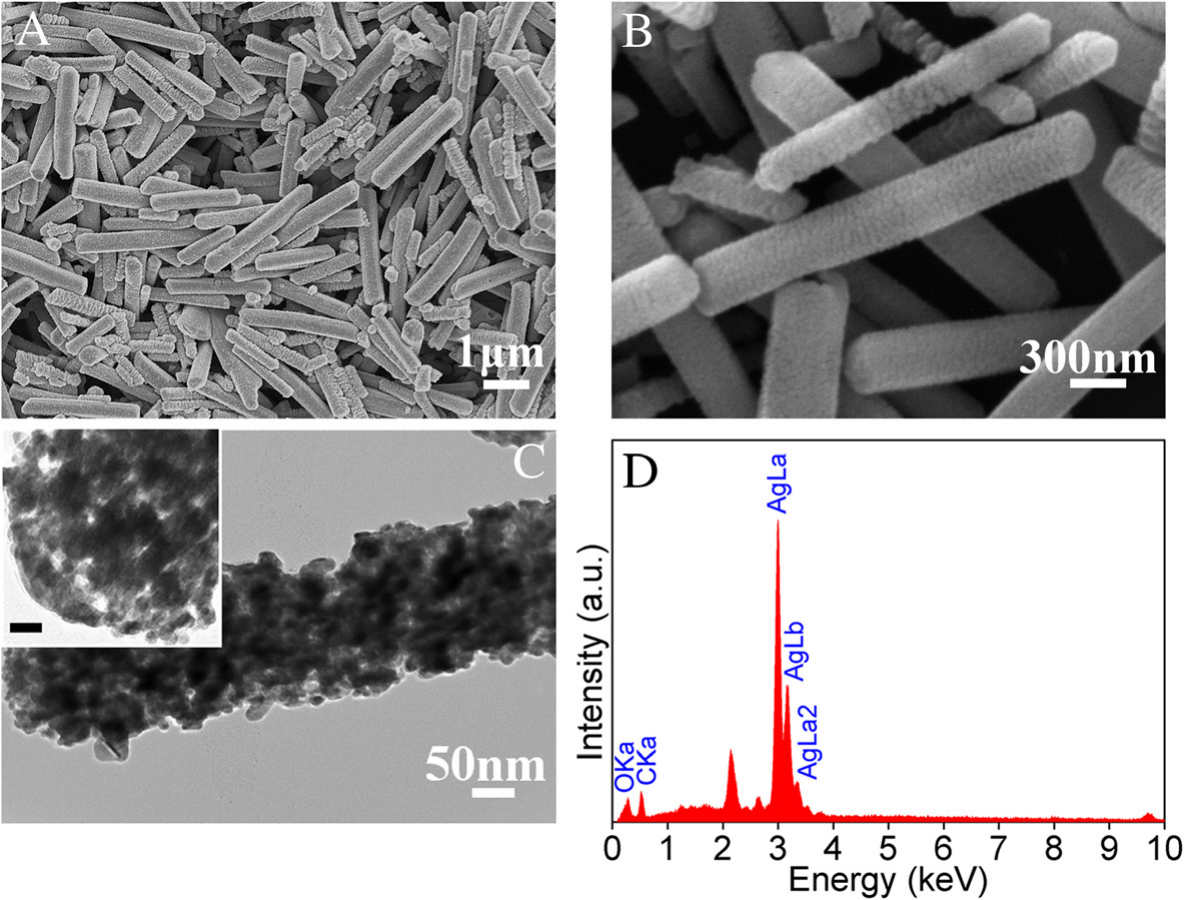
SEM, TEM images, and EDS pattern. **(A**, **B)** Low- and high-magnified SEM images. **(C)** TEM images (inset scale bar = 20 nm). **(D)** EDS pattern of as-prepared Ag_2_CO_3_ samples.

The porous Ag_2_CO_3_ nanorods were successfully synthesized by the precipitation reaction between AgNO_3_ of aqueous solution in the presence of PVP-K90 and NaHCO_3_ at room temperature. Figure 1A,B shows typical morphology of samples with different magnifications. From Figure 1A, it can be clearly seen that the products are uniformly dispersed and present nanorod morphology with length of about 3 μm and diameter of 300 nm. Moreover, the high-magnification SEM image in Figure 1B indicates that the surface of the Ag_2_CO_3_ nanorods displayed certain roughness, which implies that the nanorods are of porous feature. To get more information about the morphology and interior feature of the Ag_2_CO_3_ nanorods, the TEM technique was used to investigate of the Ag_2_CO_3_ nanorods. As shown in Figure 1C, a typical TEM image of the as-prepared products and a mass of holes are distributed on Ag_2_CO_3_ nanorods and every nanorod is assembled by many Ag_2_CO_3_ nanocrystal grains. In addition, energy-dispersive X-Ray spectroscopy (EDS) spectrum (Figure 1D) indicates that the Ag_2_CO_3_ samples only contain C, O, and Ag elements except for the elements of Au from the supports, proving that the obtained products are composed of pure Ag_2_CO_3_.

**Figure 2:**
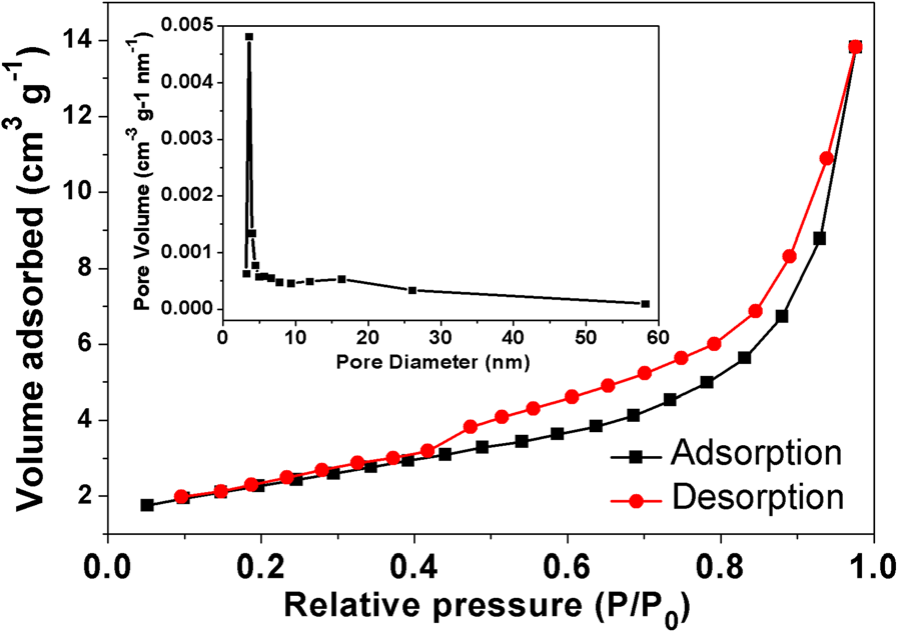
Nitrogen adsorption-desorption isotherm. Nitrogen adsorption-desorption isotherms of as-prepared porous Ag_2_CO_3_ nanorods. Insets: the pore size distribution of the crystals.

To further study the surface area and porous feature of Ag_2_CO_3_ samples, N_2_ adsorption-desorption isotherms were also measured. As shown in Figure 2, the isotherm of samples can be identified as the type IV and H3-type hysteresis loop in the IUPAC classification [29], indicating that the samples are of mesoporous feature. According to the fitting analysis with the BET equation, the surface area is 8.16 m^2^/g, which is much greater than Ag_2_CO_3_ short rods (0.91 m^2^/g) as reported in other literature [24]. Furthermore, as shown in the inset of Figure 2, the main pore size distribution is about 3.6 nm, which is good consistent with TEM observation. The formation of mesoporous is attributable to the loose aggregation of the originated nanoparticles [30,31].

**Figure 3:**
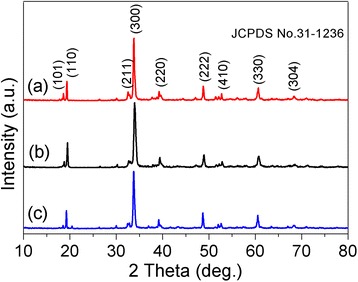
XRD patterns. XRD patterns of Ag_2_CO_3_ products prepared **(a)** with PVP-K90 as the dispersing agent, **(b)** with PVP-K30 as the dispersing agent, and **(c)** by one-time injection of the NaHCO_3_ solution.

The X-ray diffraction (XRD) was further used to characterize as-prepared Ag_2_CO_3_ samples. As shown in Figure 3, the Ag_2_CO_3_ nanorods prepared with PVP-K90 as the dispersing agent intense diffraction peaks at 2*θ* values of 17.6°, 19.3°, 32.8°, 33.8°, 39.2°, 48.5°, 52.7°, 60.4°, and 67.7° correspond to the planes of (101), (110), (211), (300), (220), (222), (410), (330), and (304). All diffraction peaks can be indexed to the typical trigonal structure Ag_2_CO_3_ crystal (JCPDS No. 31-1236) and no other diffraction peaks are detected, indicating that the obtained products are pure phase Ag_2_CO_3_ and further confirm by means of EDS characterization (Figure 1D).

**Figure 4:**
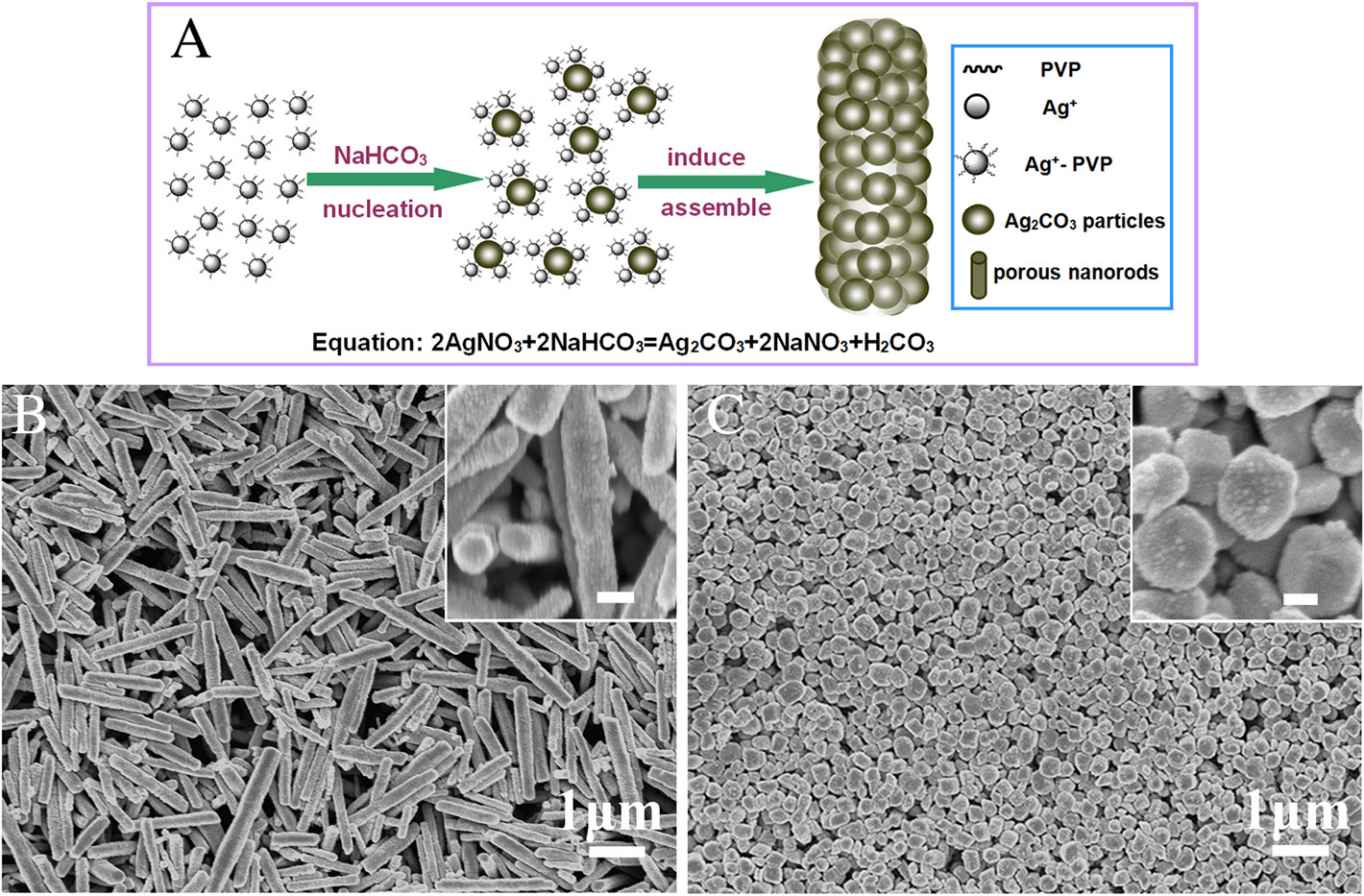
SEM images and schematic illustration of growth process. **(A)** The schematic illustration of the possible growth process from Ag_2_CO_3_ particles to porous Ag_2_CO_3_ nanorods. **(B)** SEM images of Ag_2_CO_3_ products prepared with PVP-K30 as the dispersing agent (inset scale bar = 100 nm). **(C)** SEM images of Ag_2_CO_3_ products prepared by one-time injection of the NaHCO_3_ solution (inset scale bar = 100 nm).

Furthermore, a potential reaction approach explaining the above growth process, as the schematic illustration, is shown in Figure 4A. The synthesis procedure mechanism can be understood as follows. Firstly, Ag^+^ ions and PVP molecules could form Ag^+^-PVP complex ions when the dissolution of PVP and AgNO_3_ in the deionized water under magnetic stirred with an appropriate frequency [32]. The formed Ag^+^-PVP complex nucleate with CO_3_^2−^ and quickly grew into uniform small Ag_2_CO_3_ nanocrystal grains with preferential (300) crystal plane, while the grains are difficult to continue growing because of isolation effect results from the surface cladding by PVP. As the reaction process continues, a large number of tiny grains assemble into nanorods via the induced effect of PVP long-chain molecules [33-35]. Due to the isolation effect of PVP dispersing agents and the intercrystallite void of aggregation procedure, there exists pore space among adjacent Ag_2_CO_3_ grains and it eventually evolves into the porous nanorod structure. In addition, we investigated the relationship between geometrical morphology of nanorods and the category of dispersing agents. When K30 was used in the reaction system, the Ag_2_CO_3_ nanorods obviously transform thinner and shorter (Figure 4B) by comparing with the as-prepared Ag_2_CO_3_ nanorods with K90. It is ascribed that K30 has smaller molecular weight, shorter molecular chain length, and lower viscosity than K90, thus result in a weaker adsorption and induced effect in the process of assembly. Furthermore, the adding method of NaHCO_3_ solution is also an important influence factor for the evolution of Ag_2_CO_3_ morphology. When accelerating the drop rate of NaHCO_3_ solution, the morphology of as-prepared Ag_2_CO_3_ becomes small cube-like with the size of about 200 nm; however, it still have a few short nanorods (Figure 4C). These changes may be related to exorbitant instantaneous concentration of CO_3_^2−^, which leads to Ag_2_CO_3_ explosive nucleation and rapid growth in short time. Owing to the isolation effect of PVP, the formed crystal particles spontaneously start ordered assembly as the aforementioned. However, the assembly process will terminate once CO_3_^2−^ specie completely consumed in the reaction system, and it means that the crystal particles will not continue to assemble following by two end face direction. Naturally, the morphology of Ag_2_CO_3_ samples exhibit cubic trait rather than nanorod. As a consequence, we believe that the geometrical morphology of Ag_2_CO_3_ can be regulated expediently by the design of experimental conditions.

**Figure 5:**
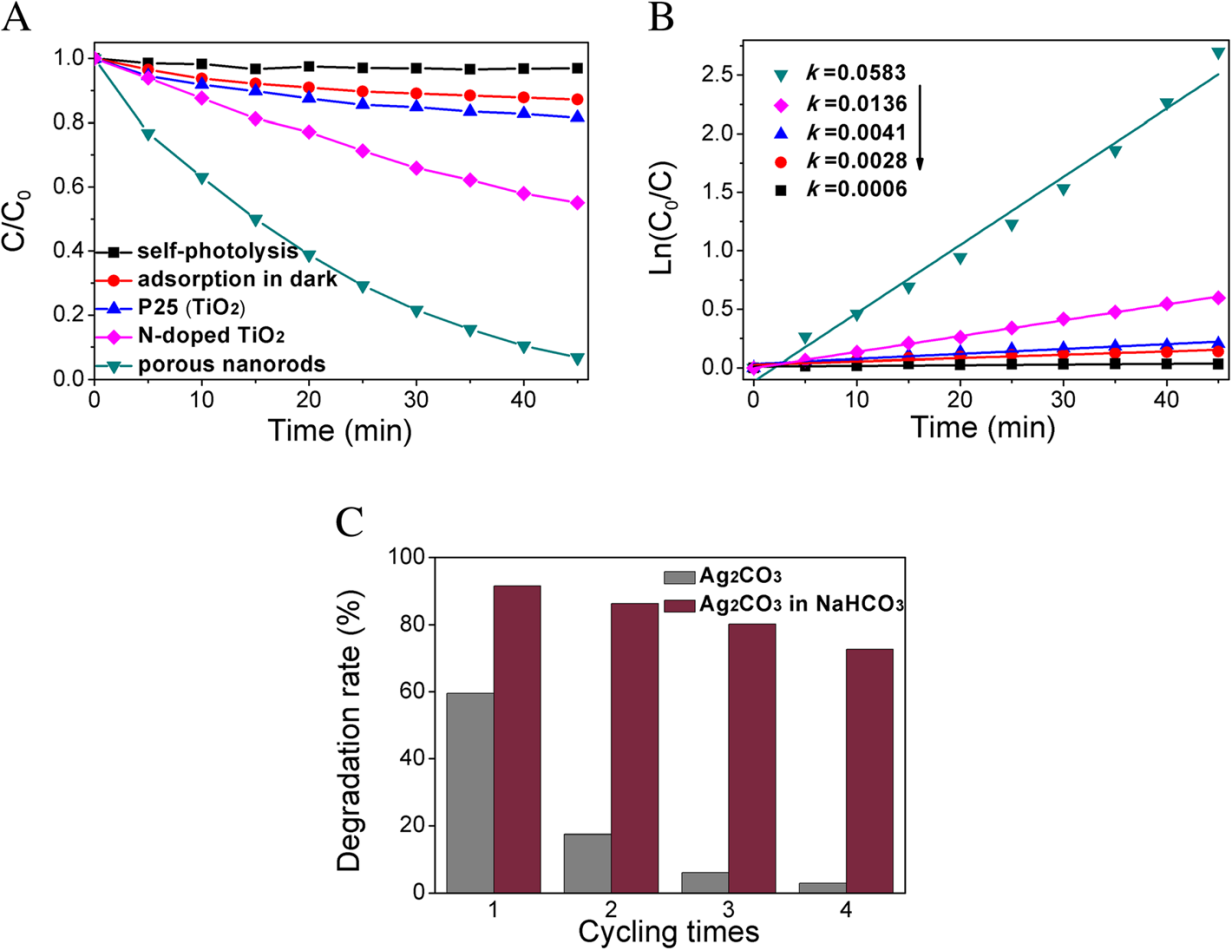
Photocatalytic degradation and stability.** (A)** Comparison of visible-light-driven photocatalytic degradation of RhB with the different samples and adsorption in the dark over porous Ag_2_CO_3_ nanorods. **(B)** Plots of ln(*C*_0_/*C*) vs. irradiation time for the degradation of RhB under visible light. **(C)** Comparison of photocatalytic stability of the porous Ag_2_CO_3_ nanorods and 0.01 M NaHCO_3_ aqueous solution in four-cycle reactions.

The photocatalytic activity of as-prepared porous Ag_2_CO_3_ nanorods was evaluated by the degradation of typical water pollutants, RhB under visible-light irradiation at room temperature. As is shown in Figure 5A, the self-degradation effect of RhB could be ignored. What is more, by comparing to the photocatalytic performance of as-prepared Ag_2_CO_3_ samples and N-doped TiO_2_ under visible-light irradiation, it indicates that the photocatalytic activity of porous Ag_2_CO_3_ nanorods is superior to N-doped TiO_2_. The degradation rate of porous Ag_2_CO_3_ nanorods can reach 94% within 45 min to RhB, while the latter only 45% in the same experimental conditions. Meanwhile, the kinetic process of the photocatalytic degradation reaction was investigated, as shown in Figure 5B. It is observed that the photocatalytic degradation reaction process follows pseudo-first-order kinetic feature with rate constant *k* of 0.0583 min^−1^ for porous Ag_2_CO_3_ nanorods, which show much higher degradation rate than the N-doped TiO_2_ and P25 (0.0136 min^−1^ and 0.0033 min^−1^, respectively). It benefits from more surface active sites and larger specific surface area for the porous structure and ultimately leads to the increase of contact area between materials and target pollution [25,26].

**Figure 6:**
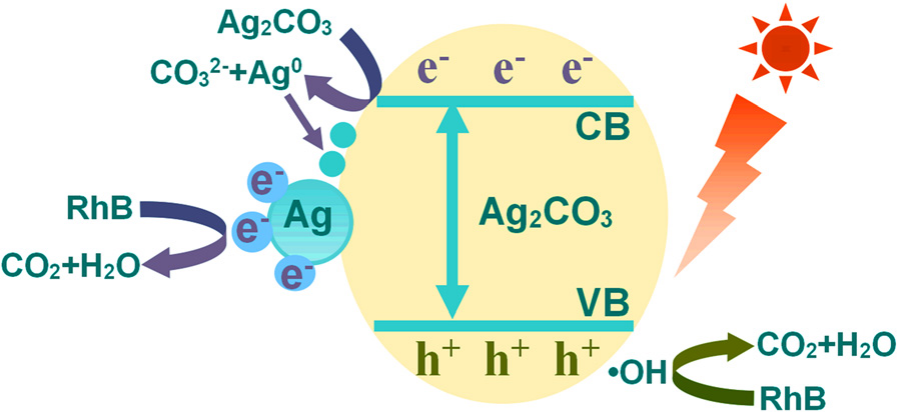
Schematic of possible photocatalytic mechanism for Ag_2_CO_3_.

The stability of the Ag_2_CO_3_ is another vital consideration except for the photocatalytic activity. It is well known that most silver compounds are light sensitive and appear metallic silver when exposed to light, including Ag_2_CO_3_ photocatalyst [22,23,36]. To evaluate the stability of the photocatalyst, the recycle experiments of RhB degradation over porous Ag_2_CO_3_ nanorods were conducted, and the result is shown in Figure 5C. It was found that after four-time cycles, the degradation efficiency of Ag_2_CO_3_ would decrease from 60% to 3%, indicating that Ag_2_CO_3_ was unstable in the absence of imperative stabilizers under visible-light irradiation. Two possible mechanisms are proposed to explain the photocatalytic activity decreased and instability (Figure 6). Ag_2_CO_3_ belongs to indirect bandgap semiconductor with a bandgap about 2.30 eV [23,24]; hence, the electrons can be effectively activated from the valence band (VB) to conduction band (CB) under visible light, leaving the holes in the valence band. The holes are capable of decomposing the pollutants in the aqueous solution. The photogenerated electrons in CB can reduce the dissolved O_2_ in the solution to form H_2_O_2_ (Equation 1) and also can reduce Ag^+^ ions in Ag_2_CO_3_ to form Ag particles (Equation 2). The formation of large amounts of Ag particles on the surface can result in instability of the Ag_2_CO_3_ under visible light. Meanwhile, they can also bring the decrease of the photocatalytic activity. In addition, Ag_2_CO_3_ has a small quantity of dissolution in aqueous solution (Equation 3, Ag^+^: 2.5 × 10^−4^ M), and the free Ag^+^ on the surface may be reduce to Ag^0^ (Equation 4). This process may further increase the solubility and promote the instability of Ag_2_CO_3_ in water.

1 $$ {\mathrm{O}}_2+2{\mathrm{e}}^{-}+2{\mathrm{H}}^{+}\to {\mathrm{H}}_2{\mathrm{O}}_2 $$

2 $$ {\mathrm{Ag}}_2{\mathrm{CO}}_3+2{\mathrm{e}}^{-}\to 2{\mathrm{Ag}}^0+{{\mathrm{CO}}_3}^{2-} $$

3 $$ {\mathrm{Ag}}_2{\mathrm{CO}}_3\rightleftharpoons 2{\mathrm{Ag}}^{+}+{{\mathrm{CO}}_3}^{2-} $$

4 $$ {\mathrm{Ag}}^{+}+{\mathrm{e}}^{-}\to {\mathrm{Ag}}^0 $$

To avoid the disadvantages of photocorrosion, we employ NaHCO_3_ as a stabilizer to inhibit the photocorrosion and decrease the solubility of Ag_2_CO_3_ in aqueous solution. So, to further evaluate the photostability of Ag_2_CO_3_ in the presence of NaHCO_3_, the recycled experiments for the photodegradation of RhB were performed, and the results are shown in Figure 5C. After four cycles, the Ag_2_CO_3_ still gives 70% degradation rate of RhB after 40-min visible-light irradiation and that the Ag_2_CO_3_ in the absence of NaHCO_3_ almost lost their activity. It indicates that the presence of NaHCO_3_ is helpful to enhance the stability and photocatalytic activity of Ag_2_CO_3_. On the basis of experimental results, two possible reasons are proposed to explain the significantly enhanced photocatalytic activity and stability of the presence of NaHCO_3_. Firstly, the NaHCO_3_ may effectively prevent the dissolution of the Ag_2_CO_3_ in aqueous solution. More importantly, when the presence of NaHCO_3_, it can facilitate reaction (Equation 2) equilibrium shift to the left and decrease photogenerated electrons reduce Ag^+^ ions in Ag_2_CO_3_. So, it avoids the formation of large amounts of Ag particles, which lead to the photocatalyst inactivate. However, a small amount of Ag particles on the surface of Ag_2_CO_3_ can become electron-rich collective. These electrons will participate in the degradation of pollutants. Thus, it promotes effective separation of electron-hole pairs.

**Figure 7:**
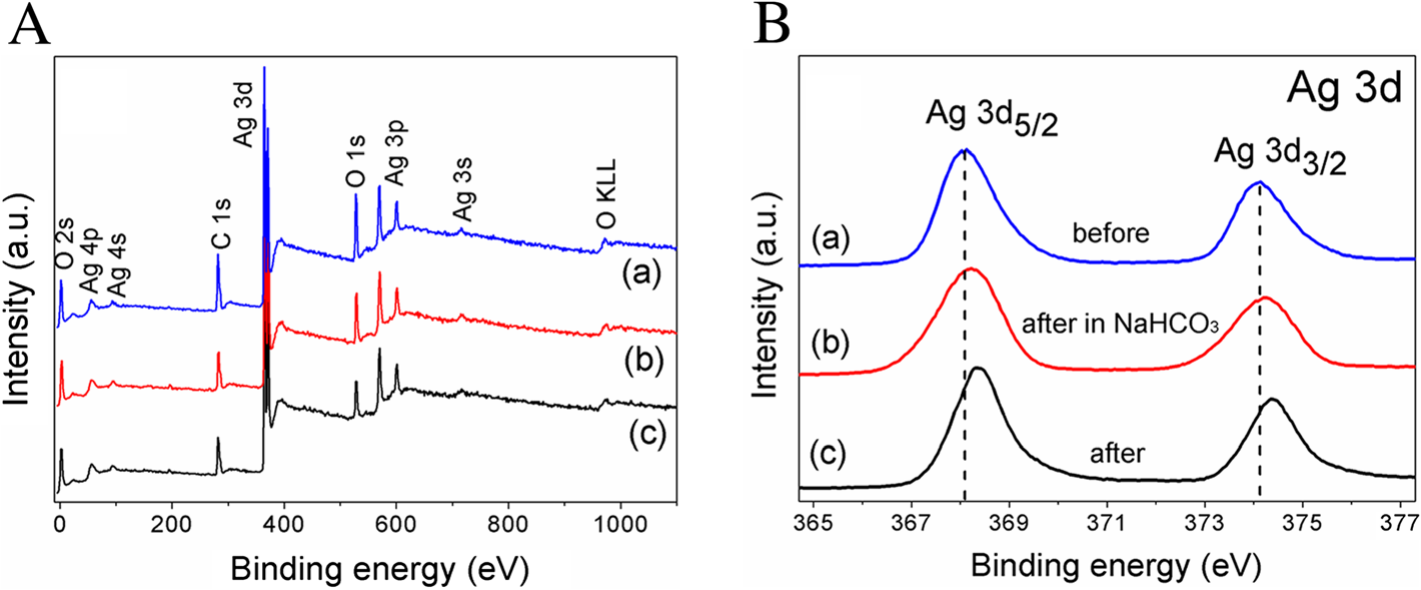
XPS spectra. **(A)** XPS survey spectra and **(B)** high-resolution XPS spectra of Ag 3d of Ag_2_CO_3_. Curves (a-c) are XPS results of Ag_2_CO_3_ before and after photodegradation experiments under visible-light irradiation.

To be more convincing of the possible mechanism, XPS measurements were performed to investigate the changes of chemical state of Ag_2_CO_3_ before and after photodegradation experiments (Figure 7). The survey XPS spectra are shown in Figure 7A. Carbon, oxygen, and silver were detected in the as-prepared Ag_2_CO_3_ samples, and no other impurities were found. Furthermore, Figure 7B shows the high-resolution XPS spectrum of Ag 3d region. As-prepared Ag_2_CO_3_ samples of Ag 3d_3/2_ and Ag 3d_5/2_ photoelectrons at 374.13 and 368.13 eV could be attributed to Ag^+^ in Ag_2_CO_3_ [37,38]. After four cycles of photodegradation experiments in the absence of NaHCO_3_, the Ag 3d_5/2_ peak obvious shifts from 368.13 to 368.33 eV, yet, the Ag 3d_5/2_ peak only shifts to 368.23 eV once the presence of NaHCO3. It is stated that a strong covalent bond between Ag^+^ cation and the ligand will result lower binding energy of Ag^+^ oxidation state than neutral Ag^0^ [39,40]. In contrast to the XPS spectra of all Ag 3d _5/2_ (Figure 7B), from curve (a) to (c), the peak shifts to the higher binding energy, which indicates the decrease of the Ag^+^ in Ag_2_CO_3_ oxidation state while the increase of Ag^0^. Meanwhile, the variation tendency confirms the restrained effect of Ag_2_CO_3_ photocorrosion in the presence of NaHCO_3_ from the perspective of experiments.

**Figure 8:**
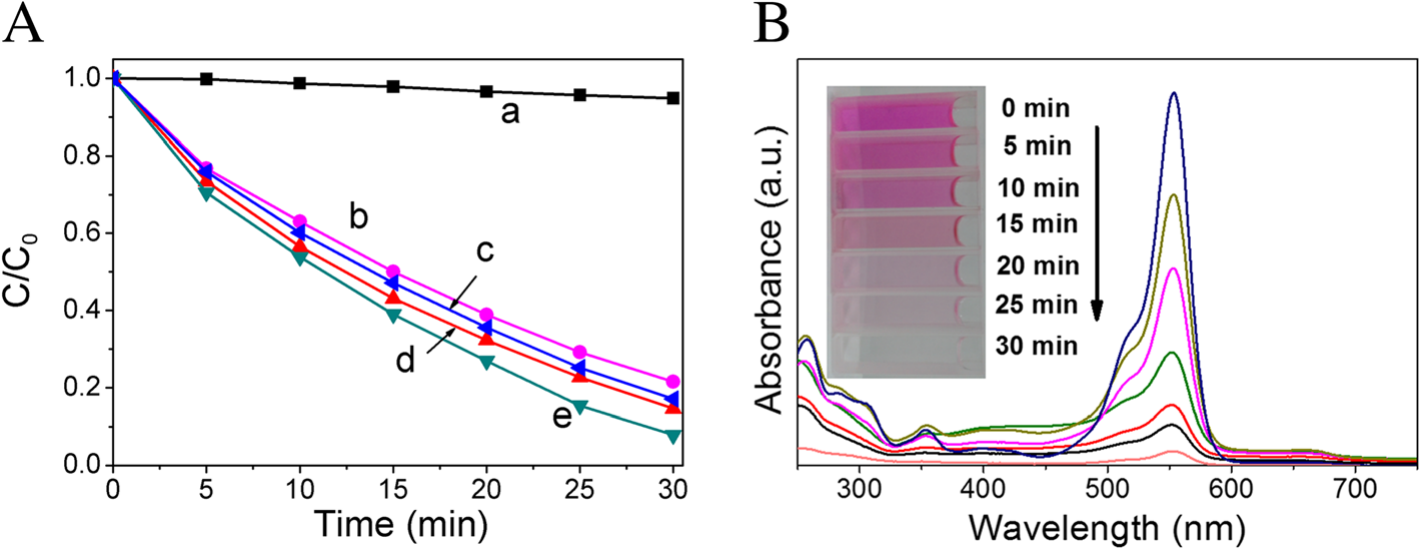
Photocatalytic degradation efficiencies and UV-vis spectral changes. **(A)** The visible-light photocatalytic activity of porous Ag_2_CO_3_ nanorods in the presence of NaHCO_3_ with different concentrations as follows: 0 M (b), 0.1 M (c), 0.001 M (d), 0.01 M (e), and 0.01 M NaHCO_3_ in the absence of photocatalysts (a). **(B)** UV-vis absorbance spectral changes of RhB aqueous in as-prepared of porous Ag_2_CO_3_ nanorods with 0.01 M NaHCO_3_ as a function of irradiation time.

Furthermore, Figure 8A shows the comparison of visible-light photocatalytic activity of porous Ag_2_CO_3_ nanorods in the presence of NaHCO_3_ with different concentrations. When only in the presence of NaHCO_3_, the RhB solutions almost were not degraded. Further observation shows that the Ag_2_CO_3_ exhibits the best photocatalytic activity in 0.01 M NaHCO_3_. When the concentration of NaHCO_3_ was reduced to 0.001 M, the degradation rate of RhB was decreased. This is due to low concentrations of NaHCO_3_ which will not effectively prevent the solubility and photogenerated electrons reduce Ag^+^ ions in Ag_2_CO_3_. However, in 0.1 M NaHCO_3_, the degradation rate is decrease even further. This may be understood as such, although excess NaHCO_3_ effectively prevents photogenerated electrons reduce Ag^+^, at the same time, the photogenerated electrons become difficult to separate from holes. Furthermore, a small amount of Ag particles can be helpful to promote photocatalytic activity. Moreover, the UV-vis absorbance spectral changes of RhB aqueous in porous Ag_2_CO_3_ nanorods in the presence of 0.01 M NaHCO_3_ as a function of irradiation time were investigated (Figure 8B). The maximum absorption wavelengths of RhB solutions are not shifting which indicate that the benzene/heterocyclic rings of the RhB molecule are decomposed [24].

## Conclusions

In summary, the novel porous Ag_2_CO_3_ nanorods were successfully synthesized by using a facile, simple, effective method. The morphology and size of the as-prepared samples can be controlled by adjusting the dispersing agent category and means of adding to reactant. The obtained porous Ag_2_CO_3_ nanorods exhibit the capability to efficiently catalyze the degradation of organic pollutants under visible-light irradiation. Furthermore, adding an appropriate concentration of NaHCO_3_ solution can effectively improve photoactivity and stability of Ag_2_CO_3_. Consequently, our work provides a one-pot aqueous solution reaction at room temperature of strategy which may be useful to extend to the synthesis of porous nanorods of other inorganic materials.
